# Screening Antibody Libraries with Colony Assay Using scFv-Alkaline Phosphatase Fusion Proteins

**DOI:** 10.3390/molecules25122905

**Published:** 2020-06-24

**Authors:** Yoshiro Hanyu, Mieko Kato

**Affiliations:** 1 Biomaterials Research Group, Health and Medical Research Institute, National Institute of Advanced Industrial Science and Technology (AIST), 1-1-1 Higashi, Tsukuba 305-8566, Japan; 2 Department of Biochemistry, Bio-Peak Co., Ltd., 584-70 Shimonojo, Takasaki 370-0854, Japan; mieko.kato@bio-peak.com

**Keywords:** antibody, single-chain Fv (scFv), alkaline phosphatase, *Escherichia coli*, screening, colony assay

## Abstract

Screening antibody libraries is an important step in establishing recombinant monoclonal antibodies. The colony assay can identify positive clones without almost any false-positives; however, its antibody library is smaller than those used in other recombinant screening methods such as phage display. Thus, to improve the efficiency of colony assays, it is necessary to increase library size per screening. Here, we report developing a colony assay with single-chain variable fragment (scFv) fused to the N-terminus of bacterial alkaline phosphatase (scFv-PhoA). The scFv-PhoA library was constructed in an expression vector specifically designed for this study. Use of this library allowed the successful and direct detection of positive clones exhibiting PhoA activity, without the need for a secondary antibody. Colony assay screening with scFv-PhoA is simple, rapid, offers a higher success rate than previous methods based on scFv libraries, and—most importantly—it enables high-throughput procedures.

## 1. Introduction

Monoclonal antibodies are indispensable for research, diagnostic, and therapeutic purposes [1,2]. To meet the demand, methods capable of rapidly generating monoclonal antibodies with high affinity and specificity are required. Furthermore, there is a need for functional antibodies with antagonistic and agonistic properties towards the target protein. To obtain antibodies with various binding characteristics, one needs to establish a large number of clones owing to the generally low percentage of positive ones. The hybridoma method is widely used for the establishment of monoclonal antibodies; however, its screening procedure is time- and labor-consuming. Moreover, the small size of the library results in an insufficient number of positive clones. In comparison, recombinant technology is more advantageous as it relies on the use of large libraries [3,4,5]. In phage display, antibodies are displayed on phages in the form of single-chain variable fragment (scFv) libraries, and the clones with antigen affinity are selected by bio-panning [6,7,8]. However, this screening method suffers from elevated rates of false-positive clones [9,10,11].

Colony assays for screening scFv libraries have been developed [12,13]. The scFv library is expressed in *Escherichia coli* bacteria, which are grown on a hydrophilic filter placed on an antigen-coated membrane. Colonies form on the filter, scFv antibodies produced by the colonies diffuse out, they bind to the antigen on the membrane, and their presence is detected by superimposing the spot on the colony. A gene encoding scFv with affinity for the antigen is obtained. The colony assay identifies clones with high reliability by directly observing antibody–antigen binding, thus resulting in a low false-positive rate. In addition, the method can be easily used to screen libraries with an order of magnitude larger (10^5^~10^6^) than those employed in hybridoma technology (10^3^~10^4^), resulting in more positive clones over a shorter period. Nevertheless, the size of a colony assay library is much smaller than that afforded by phage display (10^9^~10^10^) [3]. To obtain monoclonal antibodies with the desired characteristics, the colony assay needs to be sufficiently efficient to handle larger libraries. 

Here, we aimed to improve colony assay efficiency by replacing scFv with scFv fused to bacterial alkaline phosphatase (scFv-PhoA). The strategy of Pho-A fusion was successfully applied to produce in *E. coli* various eukaryotic molecules, including hormones [14,15] and antibody fragments [16,17,18,19,20]. All these fusion proteins were secreted in periplasm of *E. coli* where they folded correctly, yielding homogeneous, stable, and bifunctional molecules. Fusion of scFv to the N-terminus of bacterial alkaline phosphatase considerably improved performance [21,22]. The periplasmic localization of PhoA-tagged scFv ensures dimerization of the PhoA moiety into its enzymatically active form and the correct folding of scFv via disulfide bond formation [23]. PhoA enables direct enzymatic detection of scFv fusions without the need for a secondary reagent such as an anti-His-tag antibody [24]. Positive clones showing specific binding to the antigen could be detected directly and rapidly, thus strongly improving assay processivity.

## 2. Results

### 2.1. Development of the Colony Assay with scFv-PhoA

**Figure 1:**
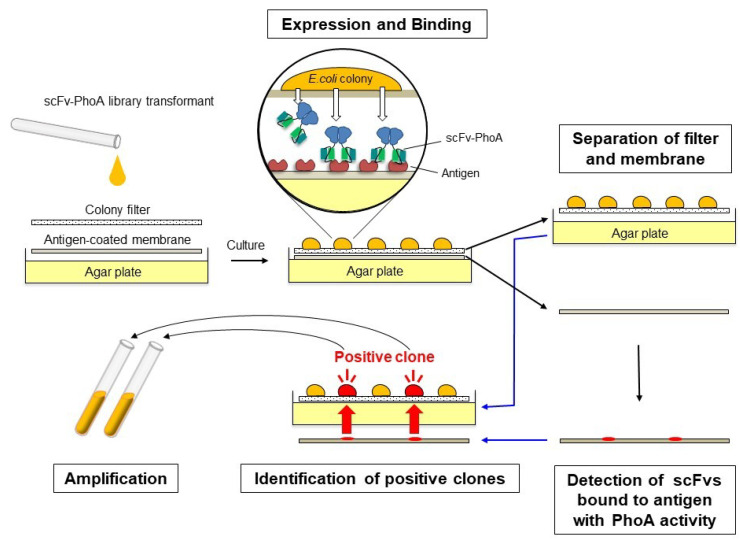
Schematic diagram of the procedure describing the colony assay with a single-chain variable fragment fused to the *N*-terminus of bacterial alkaline phosphatase (scFv-PhoA). ScFv-PhoA fusions are expressed and secreted from *E. coli*. Those with the desired affinity bind the antigen beneath the colonies and are detected directly after applying the alkaline phosphatase substrate. Positive clones are identified as the colonies matching the positive signals on the membrane.

The colony assay procedure based on the scFv-PhoA library is illustrated in Figure 1. The hydrophilic filter and antigen-coated nitrocellulose membrane were placed on the agar plate, and bacteria transformed with the scFv-PhoA library were spread on the filter. Colonies became visible after 14 h of incubation at 30 °C, at which point, scFv-PhoA expression was autoinduced. The bacterial colonies produced soluble periplasmic scFv-PhoA fusions, and those showing affinity against the antigen were captured by the antigen immobilized on the membrane. After incubation for 24 h at 30 °C, the filter harboring the colonies was transferred to a fresh agar plate and the nitrocellulose membrane was developed to detect antigen-binding scFv-PhoA fusions by chemiluminescence. This was achieved simply by applying the alkaline phosphatase substrate without using additional enzyme-conjugated antibodies and corresponding washing and reaction steps, thus shortening protocol time to about 1/10 of the original. To identify positive colonies, the filter and the membrane were superimposed so that the colonies on the filter and the positive chemiluminescence signals were aligned. The colonies corresponding to the positive signals were then picked and cultured in the medium to identify candidate genes.

### 2.2. Construction of the Expression VECTOR for Screening

**Figure 2:**
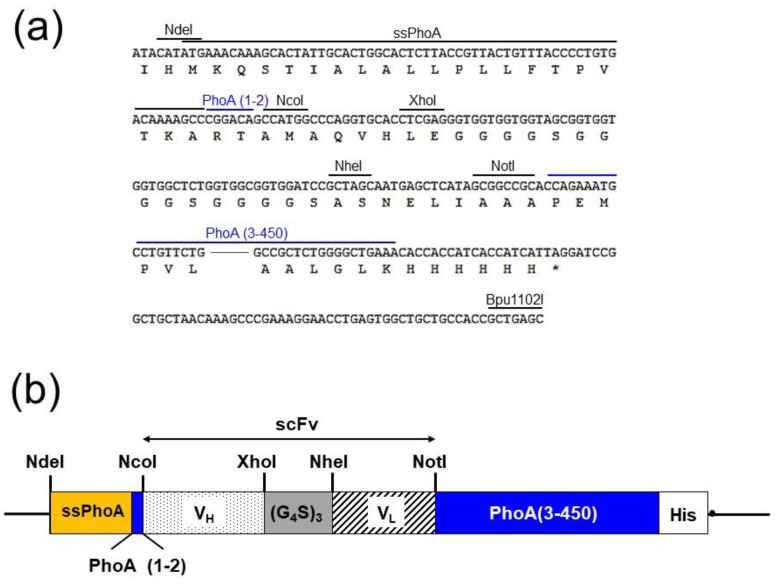
(**a**) Partial sequence of pET-NXNN-PhoA shown together with the cloning site and the restriction sites used in the cloning strategy. (**b**) Schematic representation of scFv cloned into pET-NXNN-PhoA. Orange square, signal sequence for PhoA (ssPhoA); blue square, two N-terminal amino acids of PhoA (PhoA (1-2)); dotted rectangle, heavy-chain variable domain (V_H_); gray square, linker ((G_4_S)_3_); striped rectangle, light-chain variable domain (V_L_); blue rectangle, PhoA (3-450); white square, His-tag; black circle, stop codon.

A schematic diagram of the DNA construct used for screening is shown in Figure 2 together with its restriction sites. The vector pET-NXNN-PhoA was designed and constructed based on pET-NXNN [25], which we built previously for a colony assay using scFvs and was based on pET22b (+) (Merck Millipore, Darmstadt, Germany). To allow the systematic secretion of ScFv-PhoA into the periplasmic space, we replaced the signal peptide pelB of pET-NXNN with the intact signal peptide of PhoA (ssPhoA) by NdeI and NcoI. Then, we inserted a mutated version of the bacterial alkaline phosphatase gene (*phoA*) [26] after the cloning site for scFvs by NotI and Bpu1102I. To ensure systematic processing of the PhoA signal peptide, the cloning site for scFv was located between codons +6 and +7 of the *phoA* gene [23].

### 2.3. Screening the scFv-PhoA Library

**Figure 3:**
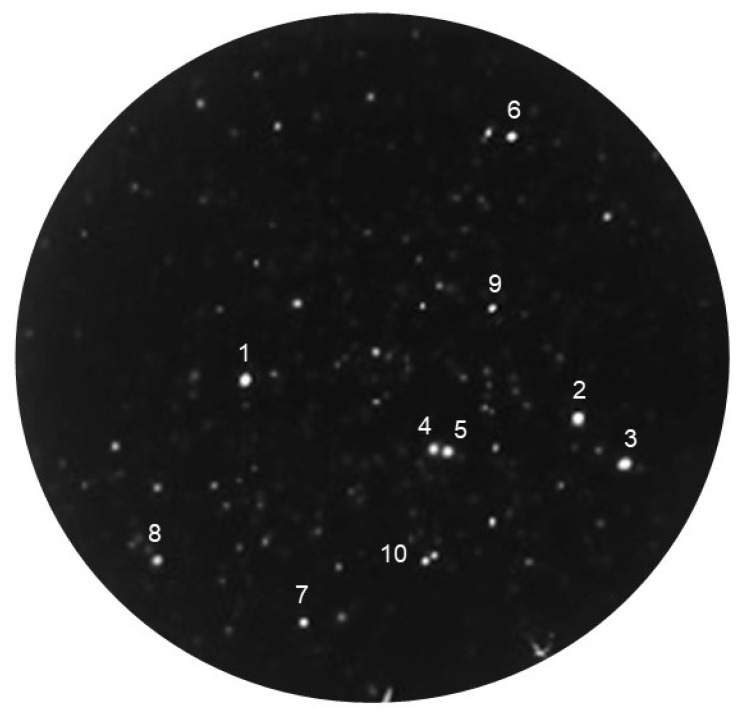
A representative nitrocellulose membrane showing positive clones from the scFv-PhoA library against human IgG. Antigen binding of scFvs to the membrane was detected by chemiluminescence. Seven positive spots that showed the strongest signal intensities were selected for further experiments.

The colony assay with the scFv-PhoA library was tested by selecting positive clones from an immune scFv library using human IgG as the antigen (Figure 3). *E. coli* were transformed with the scFv library and 3 × 10^3^ cells in a solution containing glucose and lactose were spread on a hydrophilic polyvinylidene difluoride (PVDF) filter placed on top of an antigen-coated nitrocellulose membrane on a 10 cm agar plate. Following incubation for 14 h at 30 °C, colonies with a diameter of 0.6–0.9 mm were observed. Antigen binding of scFvs to the lower membrane was detected directly by chemiluminescence in a procedure that took only 10 min per membrane, compared to the 2 h for scFvs. Avoiding secondary antibodies allowed us to reduce not only time but also background, as indicated by a higher signal-to-noise ratio of the spots compared to that of the scFv library. This, in turn, allowed the use of a lower concentration of lactose as inducer and potentially avoided the growth inhibition caused by elevated scFv expression. Formation, size, and number of colonies were observed. Spots whose intensity was above 30 (arbitrary units) were considered as positive; ten such spots can be seen in the plate shown in Figure 3. This result indicates that positive clones expressing anti-human IgG scFvs were successfully identified using the scFv-PhoA library.

**Figure 4:**
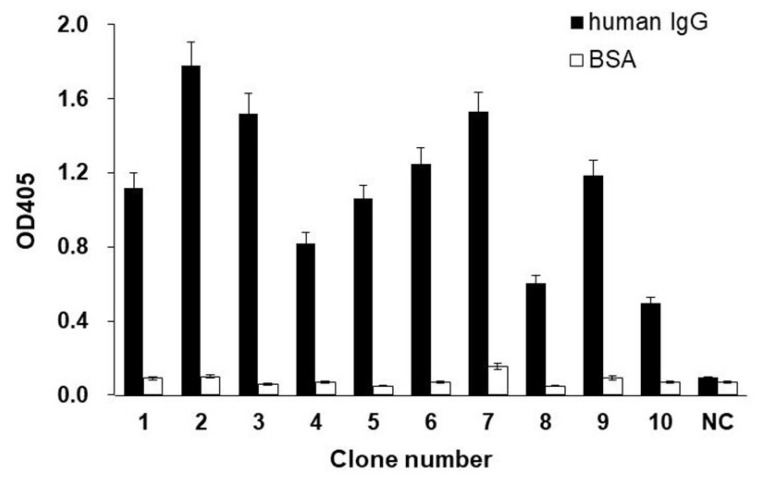
ELISA results showing the reactivity of culture supernatants from ten positive clones and one negative clone identified in Figure 3 against human IgG and bovine serum albumin (BSA; control). The negative clone (NC) was picked at random from colonies that showed no chemiluminescence signal. Data represent the mean of three replicates; error bars represent the standard deviation.

Ten clones with the strongest signal and one exhibiting no signal were selected for further analysis. Each clone was identified by superimposing the filter on the membrane chemiluminescence image. Cells were picked from the upper filter and cultured to express scFvs. The selected positive and negative clones were cultured at 37 °C in LB medium containing 50 μg/mL ampicillin until they reached OD600 of 0.6. Then, the cells were incubated at 30 °C for 6 h in the presence of 1 mM isopropyl-d-1-thiogalactopyranoside (IPTG). Thereafter, they were centrifuged at 5000× *g* and 4 °C for 5 min, and the supernatants were collected and readily examined for reactivity against human IgG using an enzyme-linked immunosorbent assay (ELISA) (Figure 4). Every identified positive clone showed antigen-specific binding activity. As a negative control, no binding was detected on the uncoated ELISA plate blocked with bovine serum albumin. Similarly, the negative clone did not bind to the antigen or bovine serum albumin. Thus, the colony assay with the scFv-PhoA library successfully established scFv-PhoA clones with binding affinity and specificity toward the antigen.

### 2.4. Sequence Analysis of Selected Clones

**Figure 5:**
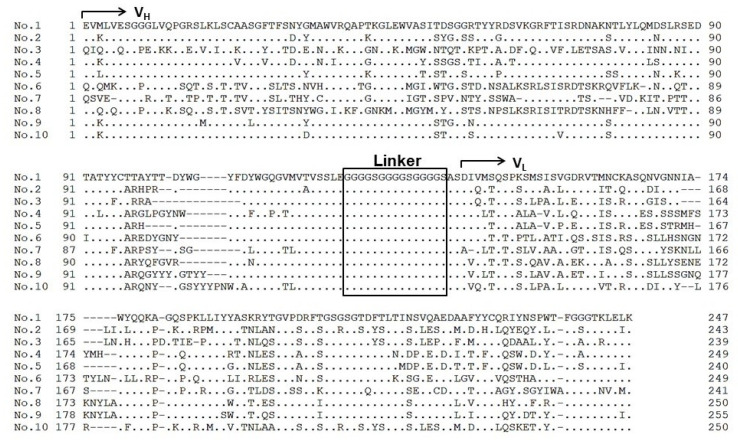
Amino acid sequence alignments of selected clones assayed. V_L_, V_H_, and linker sequences are shown. Dots and dashes indicate identical amino acids and deletions, respectively.

The DNA of each scFv from the ten clones (Figure 3) was sequenced. The inferred amino acid sequences of the ten clones are shown in Figure 5. Every clone contained the complete scFv structure consisting of heavy-chain variable domain (V_H_), linker, and light-chain variable domain (V_L_). All sequences were unique, indicating that diversity of the scFv-PhoA library was maintained throughout the assay. These results show that genes encoding scFv-PhoA fusions with binding affinity for the antigen could be successfully isolated using our method.

### 2.5. Comparison with the scFv Library

The suitability of the scFv-PhoA screening method was tested with an anti-human IgG library. Anti-human IgG scFv and scFv-PhoA libraries were constructed and transformed into *E. coli*. Transformants were spread on 10 plates (3 × 10^3^ bacteria per plate). To validate positive clones from the scFv-PhoA library, these were analyzed by colony PCR. Results from the colony assay performed with the two libraries were compared by counting the number of colonies on the filters and the spots corresponding to the positive clones on the antigen-coated membrane. As shown in Table 1, the number of colonies did not differ between the two methods, amounting to 12102 for scFv-PhoA and 11083 for scFv. The ratios of positive colonies were also similar, 1.85% and 1.75%, respectively. To validate the positive clones, these were analyzed by colony PCR and the size of the insert was checked by agarose gel electrophoresis. Positive clones with full-length scFv could be successfully obtained with both methods, and numbered 224 and 192 clones, respectively. All clones except two candidates from the scFv library presented full-length scFv inserts. The two incomplete clones were sequenced, revealing either absence of the V_H_ fragments or a shorter linker. The ratio of positively identified clones was slightly higher with the scFv-PhoA library than with the scFv library.

## 3. Discussion

The colony assay offers a superior approach for screening antibody libraries. It identifies clones by direct detection of antibody-antigen binding, thus resulting in a low false-positive rate [12,13]. Skerra et al. developed the filter-sandwich assay, in which colonies form directly on the hydrophilic filter and are then transferred to the antigen-coated membrane on the agar plate for scFv expression [27,28]. To save time, this assay omits the step whereby colonies are lifted from the agar plate to the nitrocellulose membrane. However, even like this, the procedure remains more complex than with other display methods and explains why the colony assay is not widely applied to screen antibody libraries. We previously developed a single-step colony assay that omitted the transfer of the colony filter prior to scFv expression [25]. This adjustment simplifies substantially the colony assay, reducing also contamination and cell death. To increase assay efficiency, we pioneered autoinduction of scFv expression during the assay [29]. ScFv expression represents a burden for *E. coli* growth and survival, and the induction reagent IPTG is toxic to the cells. As a result, strong induction of scFv expression occasionally leads to cell lysis and prevents rescue of the antibody gene from positive clones, decreasing overall screening efficiency. Owing to autoinduction, expression is induced at the optimal time and strength. While these improvements led to higher monoclonal antibody yields, the size of the antibody library that could be screened by this method was still much smaller than that used in phage-display [8,30]. To increase library size, here, the efficiency of the procedure was further improved by fusing scFv to the N-terminus of alkaline phosphatase, yielding scFv-PhoA.

The dimerization of alkaline phosphatase in scFv-PhoA clones has been shown to confer increased sensitivity over monomeric scFv [22]. PhoA ensures transport to the periplasmic space and the correct folding of scFv to form a disulfide bond. Fusion of scFvs to alkaline phosphatase provides a convenient, simple, and direct way of detecting antigen binding also in colony assays, substantially facilitating and speeding up the procedure [31,32]. Notably, detection of each membrane took only 10 min with scFv-PhoA compared to 2 h with scFv. Monoclonal antibodies were successfully established with this method and the percentage of positive clones was almost identical to that obtained with scFv. Hence, the rapidity and simplicity of the proposed scFv-PhoA method will make it possible to scale-up the size of the library per assay and, consequently, increase the number of candidate positive clones.

The colony assay with scFv-PhoA has additional advantages over previous iterations. By omitting the need for a secondary antibody, background chemiluminescence is substantially reduced as the signal-to-noise ratio becomes higher. Coupled with increased sensitivity of detection due to scFv-PhoA dimerization, these adjustments substantially improve assay accuracy. Moreover, the improved signal allows mild induction of expression and higher rescue yield of positive clones. Here, we could use only 0.1% of lactose as inducer, instead of 0.15% for scFv. The success rate for establishing monoclonal scFvs was slightly better than that with the preceding method.

When producing scFv in *E. coli*, expression is strongly dependent on the scFv sequence [33,34]. This variability in expression prevents efficient screening as clones with low expression and high affinity against the antigen show a weak signal, whereas clones with high expression and low affinity show a strong signal. The sequence after the signal peptides and the following few amino acids are known to be important for expression and transport to the periplasm. Here, two amino acids from the N-terminus of PhoA were used as the first two amino acids after the signal sequence [23]. Further fine-tuning could limit the observed variability in expression. Identification of optimal sequences could improve expression and colony formation in the colony assay and, consequently, increase library size per assay.

The colony assay using *E. coli* is simple and rapid owing to fast bacterial growth, thus allowing large libraries to be handled [35]. However, expression of some scFvs delays *E. coli* growth or leads to cell lysis [36,37]. In these cases, mutation or deletion of scFv genes is observed [38,39]. While this prevents cell death, it also reduces the yield of positive clones. In the colony assay, *E. coli* is exposed to inducers during detection and identification. With our method, the detection time was reduced to less than 1/10, thus decreasing exposure to inducers and the risk of cell lysis. The higher sensitivity afforded by scFv-PhoA could further reduce the concentration of inducers. Still, a quantitative analysis deserves to be performed in future studies. The reduction of the library size was observed during our screening. When 3 × 10^4^ clones were spread onto plates, we found only 1.2 × 10^4^ colonies (Table 1). Some clones with toxic scFvs would not grow and form the colonies due to the leaky expression of these scFvs. These results suggest that a tighter expression regulation, including the suppression of scFvs leaky expression events, is required to prevent the library size reduction. Furthermore, the improvement of vectors and culture conditions is also necessary to maintain the library size. Any measure that lowers the stress on *E. coli* with scFv could have a positive effect on colony assay efficiency when screening antibody libraries.

A crucial advantage of the proposed colony assay is the possibility to observe antigen binding of scFv directly in real time. Accordingly, if we quantify the signal from positive clones, we may select those with the desired characteristics already during the assay. The size and magnitude of the signal may reflect the productivity and affinity of scFvs, respectively, which are key parameters for improving the assay.

In summary, use of the scFv-alkaline phosphatase fusion protein increased screening efficiency and yield of positive clones. With greater simplicity and speed of screening, it could be possible to enlarge the size of the library per assay. This, in turn, would increase the chance of obtaining positive clones with desired characteristics. Further improvements of the colony assay are required to handle the larger libraries.

## 4. Materials and Methods 

### 4.1. Immunization

Approximately 6-week-old female Wister rats, obtained from SLC (Tokyo, Japan), were intraperitoneally (i.p.) injected with 100 μg of normal human IgG, whole molecules, purified from serum (FUJIFILM Wako Pure Chemicals, Osaka, Japan), three times once every two weeks. The first injection was in complete Freund’s adjuvant (Sigma-Aldrich, St. Louis, MO, USA), whereas the second and third were in incomplete Freund’s adjuvant (Sigma-Aldrich) in a volume of 0.2 mL. The rats were intravenously (i.v.) boosted with 100 μg of human IgG in normal phosphate-buffered saline (PBS) one week after the third i.p. injection and then sacrificed three days after the i.v. boost, following which, their spleens were aseptically removed. All animals were cared for and maintained in accordance with the guidelines of the National Institute of Advanced Industrial Science and Technology (AIST). The project was approved by the committee for Experiments involving Animals of AIST (Project identification code: A2015-033, April 2015).

### 4.2. Construction of scFv-PhoA and scFv Libraries

RNA from the collected rat spleens was purified using the NucleoSpin RNA kit (Macherey-Nagel, Düren, Germany), and the corresponding cDNA was synthesized using the PrimeScript II Reverse Transcriptase 1st strand cDNA Synthesis Kit (Takara Bio, Shiga, Japan). cDNA was used as template for PCR amplification of V_L_ and V_H_ sequences; the amplification protocol was as follows: initial denaturation at 94 °C for 2 min; 25 cycles of 98 °C for 10 s, 56 °C for 30 s, and 68 °C for 1 min; and a final elongation step at 68 °C for 2 min. The PCR was performed using KOD FX polymerase (Toyobo, Osaka, Japan) with 10 pM of the primers previously reported by Sepulveda and Shoemaker [40]. NcoI and XhoI restriction sites were added to the 5′ end of the forward and reverse V_H_ primers, whereas NheI and NotI sites were added to the 5′ end of the forward and reverse V_L_ primers, respectively. The pET-NXNN-PhoA (Figure 2) and pET-NXNN expression vectors were used for scFv-PhoA and scFv libraries, respectively. To construct pET-NXNN-PhoA, DNA fragments (Figure 2a) with signal peptide sequence (ssPhoA), linker, and restriction sites for cloning V_H_ and V_L_ fragments were synthesized by GenScript (Piscataway, NJ). The generated fragment was digested with NdeI and Bpu1102I (New England Biolabs, Ipswich, MA) and inserted into pET-NXNN (Figure 2b). Then, a mutated version of the bacterial alkaline phosphatase gene (*phoA*) [26] was inserted after the cloning site for scFvs by NotI and Bpu1102I. V_L_ fragments and the vector were digested with NheI and NotI (New England Biolabs) and purified using the NucleoSpin Gel and PCR clean-up kit (Macherey-Nagel). The V_L_ library was constructed by ligating V_L_ fragments and the vector. Chemically competent *E. coli* DH5α cells (Nippon Gene, Tokyo, Japan) were transformed with the resulting vectors. The transformed bacteria were incubated in 6 mL SOC medium for 1 h at 37 °C, after which the cells were plated on LB plates supplemented with 1% glucose and 50 μg/mL ampicillin (FUJIFILM Wako Pure Chemicals) and cultured overnight at 37 °C. The cells were then collected, suspended in 10 mL LB medium, and the vectors containing the V_L_ fragments were purified using the NucleoSpin Plasmid EasyPure kit (Macherey-Nagel). V_H_ fragments and vectors containing the V_L_ fragments were digested with NcoI and XhoI (New England Biolabs), and the scFv library was constructed by ligating the V_H_ fragments to the vectors. *E. coli* BL21 (DE3) cells (Nippon Gene) were transformed with these vectors and cultured as described above. Then, the cells were collected and suspended in 10 mL LB medium supplemented with 50 μg/mL ampicillin and used for the colony assay.

### 4.3. Colony Growth and scFv-PhoA and scFv Expression

A nitrocellulose membrane of 9 cm in diameter (Bio-Rad Laboratories, Hercules, CA) was incubated for 2 h in PBS containing human IgG (100 μg/mL). The IgG-coated membrane was then blocked for 2 h in PBS containing 5% nonfat dry milk, washed twice in PBS, and placed on a 10 cm LB agar plate containing 50 μg/mL ampicillin [29]. A hydrophilic PVDF filter of 9 cm in diameter (Durapore; Merck Millipore) was positioned atop the antigen-coated membrane. Transformed *E. coli* were grown in the above medium, and 1–5 × 10^3^ cells from the exponential growth phase were suspended in LB medium containing 0.05% glucose and 0.1% lactose. In case of the scFv library, LB medium containing 0.05% glucose and 0.15% lactose was used. The suspension was spread on the filter and incubated at 30 °C for 24 h. Colonies became visible after a 14 h incubation at 30 °C, at which point scFv-PhoA expression was induced automatically with lactose and glucose [29,41]. The expressed scFv-PhoA fusions diffused through the filter to the membrane, and those that exhibited binding affinity for the antigen were captured by the antigen immobilized on the membrane. The filter harboring the colonies was removed, placed in a fresh LB agar plate containing 1% glucose and 50 μg/mL ampicillin, and stored at 4 °C for later recovery of the bacteria. The nitrocellulose membrane was immersed in PBS containing 0.05% Tween-20 (PBS-T) and used for the detection of antigen-specific scFv expression.

### 4.4. Detection of Antigen Binding and Identification of Positive Clones

The membrane obtained after the preceding step was washed twice. The chemiluminescence signal was detected using a Chemi-Stage CC16mini (KURABO, Osaka, Japan) after development with Novex AP chemiluminescent substrate (Thermo Fishes Scientific, Waltham, MA). For the scFv-library, the membrane was incubated for 2 h with a horseradish peroxidase (HRP)-conjugated anti-His antibody (1:5000 in PBS-T; FUJIFILM Wako Pure Chemicals), and then washed extensively in PBS-T. The chemiluminescence signal was detected using a Chemi-Stage CC16mini after development with a chemiluminescent HRP substrate kit (Immobilon Western; Merck Millipore). The filter harboring the colonies and the image presenting the chemiluminescence results were superimposed, allowing the positive colonies corresponding to the chemiluminescence signals to be identified.

### 4.5. Expression of Positive Clones

The selected positive clones were cultured at 37 °C in LB medium containing 50 μg/mL ampicillin until they reached OD600 of 0.6. The cells were incubated at 30 °C for 6 h in the presence of 1 mM IPTG. They were centrifuged at 5000× *g* and 4 °C for 5 min; the supernatants were collected and were analyzed using ELISA. All experiments were conducted twice, and the average signal intensity was used in the analysis.

### 4.6. ELISA

Each well of 96-well ELISA plates was coated with 100 μL of 5 μg/mL human IgG, and then incubated with a blocking solution (Blocking Reagent for ELISA; Roche Diagnostics, Basel, Switzerland) for 2 h. The plates were washed with PBS-T, after which 100 μL of the supernatant of bacterial cultures was added to each well. The wells were washed. The amount of antigen-specific antibody present was measured using *p*-nitrophenyl phosphate (Sigma-Aldrich) and the plates were read using a microplate reader (Model 680; Bio-Rad Laboratories) at a wavelength of 405 nm.

### 4.7. Sequencing

The expression vectors were purified from positive clones. The sequences of scFvs were determined on an ABI Perkin Elmer 373A automated DNA sequencer (Applied Biosystems, Foster City, CA, USA).

### 4.8. Colony PCR of Positive Clones

For statistical validation, colony PCR was performed on positive clones from each library [42]. Anti-human IgG scFv and scFv-PhoA libraries were constructed. *E. coli* were transformed with one of the two libraries, and bacteria were spread on 10 plates (3 × 10^3^ bacteria per plate). The number of colonies and positive clones was counted for each library. Individual clones identified as positive for antigen binding were picked for colony PCR, which was carried out as follows: initial denaturation at 94 °C for 2 min; 30 cycles of 98 °C for 10 s, 63 °C for 15 s, and 68 °C for 2 min; and a final elongation step at 68 °C for 2 min. The scFvs were amplified by PCR using Hot Start Taq DNA polymerase (Takara Bio) with forward (5’-CTGTGACAAAAGCCCGGACAG-3’) and reverse primers (5’-CAGTAATATCGCCCTGAGC-3’) at a concentration of 1 μM each. The products were analyzed on a 1.5% agarose gel to check for size and purity.

## 5. Conclusions

We report the development of a colony assay for screening antibody libraries using scFv fused to the *N*-terminus of bacterial alkaline phosphatase (scFv-PhoA) and a specifically designed expression vector. The colony assay was successfully carried out with the scFv-PhoA library inserted in this vector. Positive clones could be detected based on PhoA activity, without a secondary antibody. The detection procedure was simple and rapid. The monoclonal scFvs were established with a slightly better success rate than observed when using the scFv library, and the scFv-PhoA screening procedure was validated. High-throughput screening becomes possible with the scFv-PhoA library.

## Figures and Tables

**Table 1:** Quantitative analysis of the colony-assay. *E. coli* were transformed with the scFv-PhoA and scFv libraries, and bacteria were spread on 10 plates (3 × 10^3^ bacteria per plate). Colonies growing on the filter and spots corresponding to the positive clones on the antigen-coated membrane were counted. The number of colonies that had full-length scFv inserts was determined by colony PCR.

	Number of Colonies	Number of Positive Spots	Ratio (%)	Complete scFv Insert
scFv-PhoA library	12,102	224	1.85	224
scFv library	11,083	194	1.75	192
